# Neural Network Model Combination for Video-Based Blood Pressure Estimation: New Approach and Evaluation

**DOI:** 10.3390/s23041753

**Published:** 2023-02-04

**Authors:** Batol Hamoud, Alexey Kashevnik, Walaa Othman, Nikolay Shilov

**Affiliations:** 1 Information Technology and Programming Faculty, ITMO University, St. Petersburg 197101, Russia; 2 St. Petersburg Federal Research Center of the Russian Academy of Sciences (SPC RAS), St. Petersburg 199178, Russia; 3 Department of Applied Mathematics and Cybernetics, Institute of Mathematics and Information Technologies, Petrozavodsk State University (PetrSU), Petrozavodsk 185910, Russia

**Keywords:** blood pressure estimation, neural network, computer vision, photoplethysmography

## Abstract

One of the most effective vital signs of health conditions is blood pressure. It has such an impact that changes your state from completely relaxed to extremely unpleasant, which makes the task of blood pressure monitoring a main procedure that almost everyone undergoes whenever there is something wrong or suspicious with his/her health condition. The most popular and accurate ways to measure blood pressure are cuff-based, inconvenient, and pricey, but on the bright side, many experimental studies prove that changes in the color intensities of the RGB channels represent variation in the blood that flows beneath the skin, which is strongly related to blood pressure; hence, we present a novel approach to blood pressure estimation based on the analysis of human face video using hybrid deep learning models. We deeply analyzed proposed approaches and methods to develop combinations of state-of-the-art models that were validated by their testing results on the Vision for Vitals (V4V) dataset compared to the performance of other available proposed models. Additionally, we came up with a new metric to evaluate the performance of our models using Pearson’s correlation coefficient between the predicted blood pressure of the subjects and their respiratory rate at each minute, which is provided by our own dataset that includes 60 videos of operators working on personal computers for almost 20 min in each video. Our method provides a cuff-less, fast, and comfortable way to estimate blood pressure with no need for any equipment except the camera of your smartphone.

## 1. Introduction

Blood pressure is considered one of the most significant vital signs, and it impacts the health state directly since high blood pressure is one of the most serious risk factors for cardiovascular disease (CVD), which is the leading cause of death in many cases [1]. Based on this fact, blood pressure monitoring became an essential procedure that must be performed periodically. On the other hand, the most common methods for accurately measuring blood pressure are cuff-based, expensive, and cumbersome and they should not be used frequently without a doctor’s supervision [2]; therefore, noncontact blood pressure sensing has received a lot of recognition recently because it enables simple, convenient, and nonrestrictive estimation of blood pressure. One of the techniques to achieve noninvasive measuring of vital signs is a photoplethysmogram (PPG), which measures volumetric variations in blood circulation using a light source and a photodetector to measure color variations at the skin’s surface [3,4]. Derived from the fact that the changes in the color intensities of the channels carry information about variation in the blood that flows beneath the skin, we propose our novel approach to estimate blood pressure by using only a video of the subject’s face. Our approach is based on cropping regions of interest (ROIs), which are the portions of the image (frame) that we want to operate, and feeding them directly into a convolutional neural network (CNN) [5], which is a type of artificial neural network that can automatically learn and extract the features of each frame channel followed by long short-term memory (LSTM) [6], which is a type of recurrent neural network (RNN) that is able to learn long-term dependencies, especially in sequence prediction problems. We used LSTM to learn how the changes in the intensities throughout the recording duration lead us to estimate systolic and diastolic blood pressure (SBP and DBP, respectively). We intended to make our approach simple, convenient, and straightforward without any need for equipment other than the camera. It can be used for scenarios such as vehicle driver monitoring or operator monitoring where it is not convenient to attach special devices (e.g., electrocardiography (ECG) monitor) to a person. Due to this fact, we emphasized designing a cuff-less, contactless, and comfortable approach that depends only on image analysis. In other words, our method is inexpensive, manageable, and distinguished from other previous methods since it does not need to extract any signal from the face or any other regions and it does not use any additional information from electrocardiogram (ECG) and arterial blood pressure (ABP) signals, which contain information of cardiac status; hence, our approach is less complex and time consuming than other techniques.

The rest of the paper is organized as follows. Section 2 contains an overview of existing approaches and methods that are proposed to achieve noninvasive estimation of blood pressure. Section 3 consists of two main subsections; the first one includes information about the datasets we used, whereas the second one contains all the details about our experiments on building, training, and testing our models. Section 4 shows the results of testing our models and comparing them with other published models. Section 5 discusses our approach and the results with the limitations of our technique. The paper finishes with Section 6, which includes the conclusion and future scope of the proposed approach.

## 2. Related Work

Recent years have seen a substantial increase in interest in blood pressure estimation using extracted remote PPG (rPPG), which is an advancement of PPG technology that does not require physical touch and instead relies on ambient light reflected from the skin and collected remotely by a complementary metal–oxide–semiconductor (CMOS) camera followed by supervised machine learning algorithms [7]; therefore, this section includes outlines of the recently proposed methods and algorithms to accomplish cuff-less prediction of blood pressure using videos of the subject.

Luo et al. [8] used transdermal optical imaging (TOI) to process imperceptible facial blood flow changes from 17 different ROIs in the face followed by an advanced machine learning algorithm. They managed to score a mean error + SD of 0.39 ± 7.30 mmHg for SBP and −0.20 ± 6.00 mmHg for DBP, while the mean accuracy was 94.81% and 95.71% for SBP and DBP, respectively. Jain et al. [2] assumed that any intensity variation observed in the red channel should be due to variation in the blood that flows beneath the face skin under constant lighting conditions and camera settings throughout the recording; hence, they used principal component analysis (PCA) to extract these variations from the video. They used the detected peaks of the preprocessed signal to extract the time and frequency domain parameters, based on which SBP and DBP were estimated using a linear regression model. Their model achieved mean absolute errors (MAEs) of 3.9 mmHg and 3.7 mmHg for SBP and DBP, respectively.

Other approaches such as that of Secerbegovic et al. [3] used the pulse transit time to estimate blood pressure after applying independent component analysis (ICA) [9] on the raw source signals extracted from the forehead; their linear regression model achieved a total mean average error (MAE) of 9.48 mmHg for SBP and 4.48 mmHg for mean arterial pressure. ICA and linear regression were also used in [10], where Oiwa et al. tried to increase the accuracy of the estimated blood pressure by using ICA for processing the RGB signals of five obtained ROIs and by using facial PPG amplitude and nasal skin temperature as inputs of linear regression models, which predicted blood pressure with an MAE within the range of 1.5–4.5 mmHg and 1.72–4.75 mmHg, respectively.

On the other hand, using a convolutional neural network may look reasonable since we are dealing with videos and images, and that is what Iuchi et al. [11] proposed to use the spatial description of the subject’s face as an input of their model by extracting time–space information of pulse waves on the face. They managed to predict SBP and DBP with MAEs of 6.7 mmHg and 5.4 mmHg, respectively. Another interesting approach was mentioned in [12], where Wu et al. proposed a blood pressure estimator based on two-channel rPPG signals of the upper and the lower face obtained by chrominance-based (CHROM) [13] rPPG extraction besides a generative adversarial network [14] in order to get over the obstacle of the lack of data. In addition, they came up with the idea of having multiple models, each one corresponding to small nonintersecting intervals, and used a combination of age and body mass index (BMI) of the subject to choose the appropriate model to estimate the blood pressure. Schrumpf et al. [15] adopted different neural network architectures such as AlexNet [16], Resnet [17], and the architecture published by Slapnicar et al. to predict blood pressure (BP) values [18]. The training stage had multiple phases in order to find a suitable strategy to crop the signal into windows followed by transfer learning to train the models on rPPG signals extracted by the plane-orthogonal-to-skin (POS) algorithm [19]. As a result, the ResNet model achieved the lowest SBP MAE of 13.02 mmHg, while AlexNet had the lowest DBP MAE of 8.27 mmHg.

Some researchers studied the correlation between blood pressure and image-based pulse transit time (iPTT), which is calculated as a time lag between two rPPGs obtained from simultaneous recordings of two body locations. These experiments were for the purpose of extracting features that may work efficiently in the task of predicting blood pressure. Jeong et al. [20] observed that SBP has a strong correlation with the extracted PTT from the green color intensities of the regions of interest (face and palm) through videos obtained by a high-speed camera (420 fps). After some years, the same authors reimplemented their methodology in [20] but with an infrared light source along with a high-speed camera [21]. In addition, they summated the red component pixels of the regions of interest, which were detrended, filtered, and differentiated to find the correct maximum derivative points of the photoplethysmogram to obtain relevant information for BP estimation.

Other papers used other equipment to obtain the PPG signal to estimate blood pressure, as in [22], where Gaurav et al. used a PPG sensor of Samsung Galaxy Note 5. The signals were preprocessed over many stages to extract 46 features in total that were fed into three weighted artificial neural network (ANN) regression models to determine DBP, which was used as an input of three other weighted regression models besides the aforementioned 46 features in order to predict SBP. Their models achieved an MAE of 4.47 mmHg for SBP and 3.21 mmHg for DBP.

Finally, some authors considered this task as a classification problem instead of regression, and an example of this approach is what Visvanathan et al. [23] worked on. They used demographic features such as height, weight, and age besides 14 extracted features from the PPG signal to improve the accuracy of the predictions with both linear regression and SVM algorithms, which classified the output into five categories (from very low BP to very high BP) and achieved accuracies of 100% and 99.29% for SBP and DBP, respectively.

Since it is obvious that most papers are focused on the derivation of pulse waves taken from one or more regions of the subject’s face, we developed in this paper a novel method for blood pressure estimation with no need to extract any signal from the face or any other region; we propose a less computationally costly and time-consuming approach that predicts blood pressure by feeding images of regions of interest cropped from each frame of the video into our models, which have special architecture (CNN + LSTM + fully connected layers). In addition, we mention that our approach does not need any auxiliary signals such as ECG or developed equipment except a smartphone or any device that has a camera.

Since none of the papers we included above worked with raw frames without the preprocessing or extraction of signals, we had to investigate and find suitable models to compare our models with to prove the validity of our work. After searching and exploring, we accessed the models mentioned in [15], and we managed to apply the adopted signal extraction method on the datasets used in this paper followed by testing their models to compare their performance with the results we achieved by our models on the same test set.

## 3. Materials and Methods

This section is divided into two main parts: datasets and the proposed approach. In the Datasets subsection, we overview the used datasets for training and testing our models, whereas the Proposed Approach part includes the details of building our models starting with fundamental decisions about ROIs and architecture selection and concluding with testing them according to basic and novel standards.

### 3.1. Datasets

In this subsection, we include two datasets that we dealt with during our work procedure. The first dataset is the Vision for Vitals (V4V) dataset [24,25], which we used to train our models to accomplish noninvasive estimation of blood pressure. The second dataset is our own dataset, which provides the subjects’ respiratory rate at every minute and which we used to create new a criterion to test the validity of our models as we explain later.

#### 3.1.1. V4V Dataset

In order to train our models, we need a dataset consisting of videos of subjects looking at the camera so their faces can be detected and captured during the whole video. In addition to the videos, the values of their SBP and DBP should be available at each second. V4V dataset contains 724 videos in total (25 frames per second) of 100 subjects, where each subject has between 1 and 10 records with different durations. The dataset also includes text files, each one of which is related to a specific video. These files contain raw blood pressure signals with a frequency of 1 kHz.

According to the National Library of Medicine, when the heart beats, the heart muscle contracts and pumps oxygen-rich blood into the blood vessels, creating SBP. Conversely, DBP is the pressure on the blood vessels when the heart muscle relaxes [26]; hence, SBP and DBP can be extracted by observing the peaks and valleys of the blood pressure signal, which was performed by a simple code written in Python 3.8 using SciPy library (access date: 28 September 2022). We processed the output to represent the blood pressure at each second, which is the purpose of this paper.

**Figure 1:**
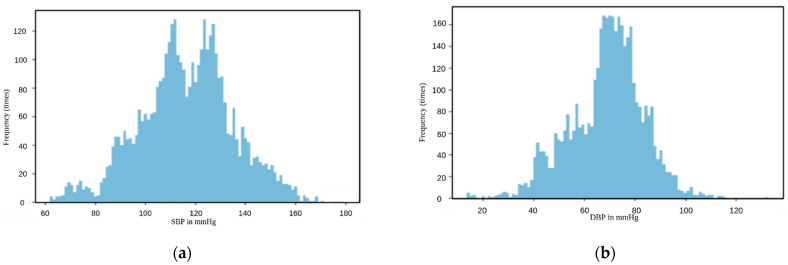
Distribution of the samples before upsampling: (**a**) distribution of SBP samples before upsampling; (**b**) distribution of DBP samples before upsampling.

**Figure 2:**
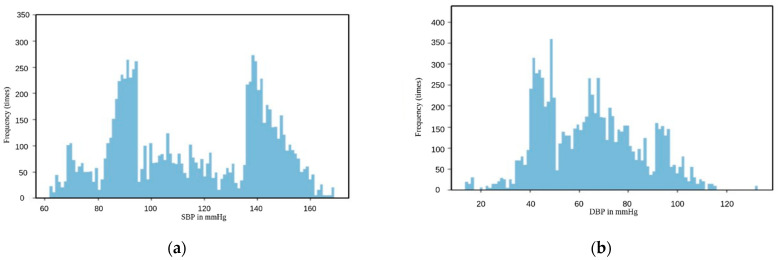
Distribution of the samples in the training sets after upsampling: (**a**) distribution of samples used to train models to estimate SBP; (**b**) distribution of samples used to train models to estimate DBP.

Regarding the training, validation, and testing sets, 140 videos were used (divided into 70% training, 20% validation, and 10% testing) to ensure that we cover most of the subjects and to include the majority of SBP and DBP values without redundant repetition of the usual or common values that represent the normal state of the subjects, since most of the are young and it is reasonable to have blood pressure within the normal range. Based on the distribution of the samples in Figure 1, it is obvious that the dataset had a normal distribution, which, according to our knowledge, may cause overfitting and lead the models to always predict the blood pressure value from the interval that contains the most repeated values. Therefore, we were trying to achieve a kind of uniform distribution so that our models are not biased. We decided to upsample the bands of frames from the whole dataset that present unusual values of SBP and DBP because we focused on only the uncommon values. Some subjects had common and uncommon values in the same video; that is why we did not upsample the subjects because, by doing this, we obtain a higher frequency of the usual values as well as the uncommon ones and the whole process of upsampling becomes useless. The number of frames was 174,625 before the upsampling, while it went up after the upsampling to 323,825 frames, and the new distributions are shown in Figure 2. However, we were not able to achieve a uniform distribution because high SBP sometimes occurs with normal DBP, and low DBP may come with normal SBP [27]; hence, while we were duplicating the frames that represent uncommon SBP, some of them represented normal DBP and vice versa, so upsampling did not give the result we were hoping for, but it was still better than training the models over pure normal distributions. Therefore, we decided to stop the upsampling when we obtained a sufficient number of samples from each range (low, normal, and high), so the probability of having biased models in some specific range is unlikely.

#### 3.1.2. Operator Dataset

We introduce our own dataset, which is called the Operators dataset. It includes 60 videos of operators sitting in front of their own computers reading articles or working. The videos were taken over mostly consecutive days 2 or 3 times a day to cover the morning and evening periods (sometimes, the afternoon period is included).

The durations of the videos are between 16 and 20 min with a resolution of 640 × 480 for all videos but, on the other hand, 58 videos have a frame rate of 30 fps, whereas one video has a frame rate of 7 fps, and another one has a frame rate of 15 fps. Moreover, the ground truth of the respiratory rate of the subject is available for each minute.

Due to the difference between the frame rate of these videos and the input of our models (25 frame per sample), we tested three methodologies to choose the frames to be the input of the models. The methodologies were to take 25 random frames out of 30 frames per second, to exclude each sixth frame after every five consecutive frames, or—as the final option—to take the first 25 frames out of 30 frames per second. After the testing procedure, which will be explained in Section 3.2.4., we found that there is no significant difference between the results no matter which methodology was selected; hence, we decided to exclude each sixth frame after each cascade of five frames, which was the second method.

Regarding the video that has a frame rate of 7 fps, we decided to implement duplication for the first four frames four times and three times for the last three frames, whereas we dealt with the video that has a frame rate of 15 fps by duplicating the first ten frames and joining them to the last five frames to obtain twenty-five frames per second.

It is scientifically proven that blood pressure decreases during deep breathing, which indicates a low respiratory rate, whereas one of the earliest symptoms of high blood pressure is shortness of breath or high respiratory rate [28]. In other words, there is a proportional relationship between respiration rate and blood pressure; therefore, we came up with the idea of testing the presence of a correlation between the blood pressure predicted by our models and the provided respiratory rate as a new criterion to find out whether our models respond effectively to the changes in the subject’s respiratory rate.

### 3.2. Proposed Approach

When we look deeply into our task, we find that it consists of two main parts: feature extraction and sequence modeling based on changes in the red green blue (RGB) channels’ intensities of consecutive frames due to variation in the blood that flows beneath the face skin; therefore, we adopted a combination of a CNN and LSTM to estimate the SBP and DBP of the subjects. CNN extracts high-level spatial features, which are basically abstract representations of the parts that carry information in the image of the ROI, whereas the LSTM is responsible for temporal features, which represent the changes in the spatial features over time.

#### 3.2.1. CNN Models Selection

Training a new deep learning model from scratch necessitates a massive amount of data, powerful computational resources, and hours, if not days, of training time. In addition, collecting and annotating huge amounts of domain-specific data is time consuming and expensive, leading to the idea that implementing deep learning models is challenging, and as it is known, the knowledge of previous objects assists in learning new objects due to their similarity with the latter; hence, CNN models trained on a specific dataset or task can be fine-tuned for a new task [29]. Based on the aforementioned facts, transfer learning was included in our work for the feature extraction part. There were many candidates of pretrained models to use, each with its own characteristics and distinctive features (e.g., Resnet overcomes the vanishing gradient problem in deep architectures by virtue of their skip connections [17]). By exploring the properties of pretrained models on the Keras official website [30], we decided to go with the models that have a reasonable number of parameters and accepted depth in addition to considering the time (ms) per inference step and top 5 accuracy. The used models in this paper are Xception, DenseNet121, VGG16, Resnet50V2, InceptionV3, EfficientNetB0, EfficientNetB1, EfficientNetB2, EfficientNetB3, EfficientNetB4, and EfficientNetB5).

#### 3.2.2. ROI Selection

At the beginning of the task, determining the best ROI to use was tricky since most of the papers used different ROIs as mentioned in the related work section. Therefore, we were not able to choose the best ROI to extract. Furthermore, the number of pretrained models to use was relatively high; consequently, we decided to test these models on images of the most common ROI used previously (i.e., forehead, right cheek, and left cheek) so that we can exclude some of the candidate pretrained models and keep others based on the performance. We used the facial landmarks obtained by applying Face Alignment in Full Pose Range: A 3D Total Solution 3DDFA framework [31] to segment the faces of our subjects and extract the forehead, right cheek, and left cheek regions. The preprocessing step of the images consists of resizing the images to (32x32x3) and applying per-channel standardization of pixels by subtracting the mean from each pixel followed by division by the standard deviation.

**Figure 3:**
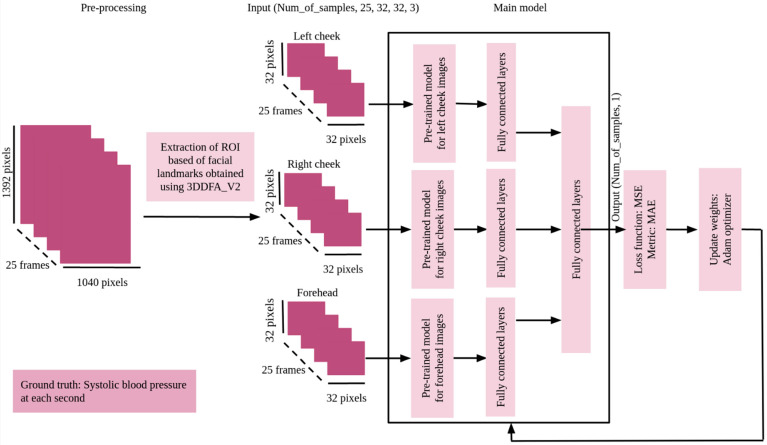
General scheme of the process of choosing CNN models.

The procedures of obtaining the ROI and training the models are time consuming, and since this step was transitional, we applied ROI extraction in this stage on 90 videos out of the final 140. We created eight models with different pretrained models followed by the same architecture of fully connected layers as Figure 3 shows and trained them over 20 epochs each. We chose Adam as an optimizer with a learning rate of 0.0001. Our loss function and metric were mean squared error (MSE) and MAE, respectively.

According to the results listed in Table 1, we found that Resnet50V2 and EfficientNet (B0, B1, and B2) had the best performance among other pretrained models (lowest root mean square error (RMSE) and MAE). Next, we were curious to find which region of interest had the most significant impact on the accuracy of the predictions in order to reduce the complexity of our models. Considering the result of the previous stage, three identical models with EfficientNetB3 as a feature extractor were created, each one fed with consecutive images of only one of the regions of interest. The training process was applied with the same hyperparameters, loss function, optimizer, and metric from the previous step. The result of this procedure is shown in Table 2, and it is obvious that feeding both cheeks to the model leads to better predictions and higher accuracy.

Therefore, based on what we obtained so far, the forehead region was excluded as an ROI, and Resnet50V2 besides EfficientNet (B0, B1, and B2) with its different versions were the chosen pretrained models to use as feature extractors in the first phase of estimating the SBP and DBP of the subjects.

#### 3.2.3. Building Our Models

**Figure 4:**
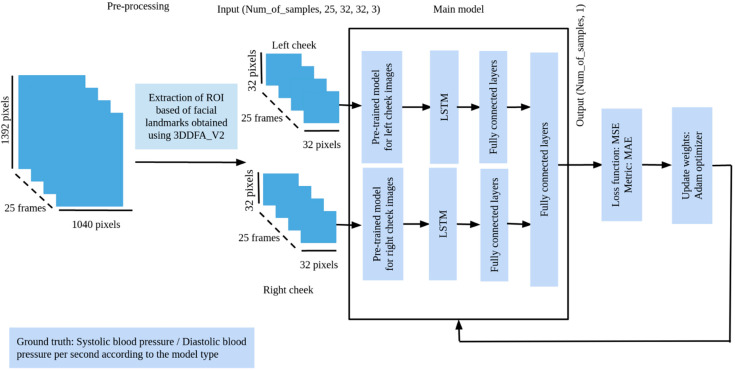
The overall structure of the training process.

We adopted the model architecture shown in Figure 4. Therefore, two pretrained models were included in the feature extraction stage, and each one of them was fed with one of the ROI images after applying the preprocessing steps mentioned in Section 3.2.2. Several experiments were carried out in this part, where we used either a different or the same pretrained model for both positions. The idea of using LSTM was to teach our model how the changes in blood volume beneath the face skin through time, which are represented by the changes in features obtained from the CNN part, have a direct effect on the predictions.

Due to many medical reasons, high SBP does not mean having high DBP and vice versa. To be specific, sometimes, a normal DBP comes with elevated SBP due to age-related stiffening of the aorta or other medical conditions [27]. Due to this fact, we decided to train two types of models, one type to predict SBP and the other one to estimate DBP, since we were concerned about the inaccurate relation between SBP and DBP that the model would come up with due to using the same features with shared weights in most layers. We had to implement this idea because generalized estimations and good performance are needed not only on this specific dataset that consists mostly of young subjects but also in real life, where any medical condition can happen.

The training/testing process was implemented with 140 videos, divided into 98 videos for training, 28 videos for validation, and 14 videos for testing, including 323,825 frames (12,953 s) in total after the attempt of balancing the dataset. The number of LSTM units is 25, and we chose this number to keep the computation cost of the model acceptable. The loss function is MSE, while the used metric is MAE. Regarding the optimizer, we chose Adam with a learning rate of 0.0001.

#### 3.2.4. Testing on Operator Dataset

The dedication in testing our models on the Operator dataset is to make sure that the accuracy of their estimations does not decrease due to the changes in the lighting conditions or the resolution of the video. We used this dataset despite the fact that the ground truth of blood pressure is not available for two reasons: the first and obvious cause was the difficulties in finding an available and not confidential dataset that is similar to the V4V dataset. The other reason was to find a new standard that we can rely on to check if the model responds effectively to the changes in RGB intensities through time, which correlates with the respiratory rate [28] that is provided at each minute. In order to accomplish this testing process, we tested our models over the 60 videos of the Operators dataset, obtaining the value of SBP or DBP (based on the type of the model) at every second. The values that present the SBP or DBP for each minute were calculated by averaging every consecutive 60 values predicted by our models, and then Pearson correlation coefficients [32] which measure the correlation between the predictions of our models and the respiratory rate, were checked and compared with the ranges of strong and weak correlation.

#### 3.2.5. Comparing with Other Models

**Figure 5:**
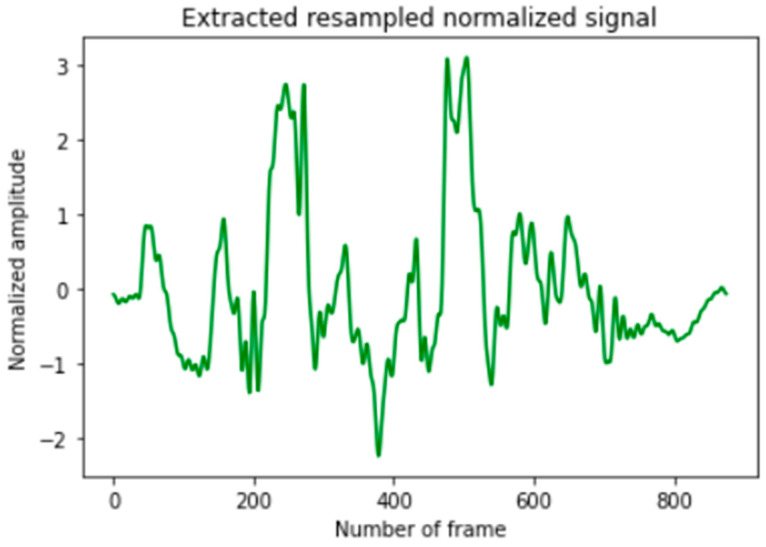
An example of extracted normalized signal from a random 7 s video using POS algorithm.

The models of [15] were available on the author’s GitHub page [33] “Fabian-Sc85/non-invasive-bp-estimation-using-deep-learning. Available online: https://github.com/Fabian-Sc85/non-invasive-bp-estimation-using-deep-learning#1 (accessed on 24 October 2022)”. There were several stages to apply on the test set to be compatible with the input form of these models. The extraction of the signal from the videos was executed as it was mentioned in their paper [15] starting with implementing the POS algorithm [19] to obtain the pulse signal, and then this signal was broken down into intervals that represent each consecutive 7 s. These intervals (called windows) were tested and excluded if their SNR is below −7 dB. According to [15], the idea of choosing this SNR was finding a tradeoff resulting in a sufficiently large number of training examples with an acceptable SNR for rPPG. However, SNR was calculated using SciPy library in Python 3.4 (access date: 27 October 2022), which returns the mean to standard deviation ratio, and then it was converted to dB. Finally, these signals were resampled and normalized to zero mean and unit variance. According to [33], the input should be 875 samples (corresponding to 7 s using a sampling frequency of 125 Hz), and since the fps of V4V dataset videos is 25, we obtained a signal of 175 samples to present 7 s, and then we had to resample it using SciPy library in Python 3.8 (access date: 28 October 2022) followed by normalization as shown in Figure 5.

These stages were the reason to exclude many videos from the test set, which we will call “V4V test set 1”, because many videos included windows with SNRs below −7 dB. Therefore, we needed to create a new test set, which will be called “V4V test set 2”, that contains new videos from the V4V dataset and shows subjects that the models never saw before during the training stage, and we focused on including the unusual values of the SBP and DBP to ensure that the result of the comparison was reliable.

Regarding the correlation with respiratory rate, the aforementioned compatibility process was applied to the videos of the Operator dataset. There were windows with SNRs below −7 dB but, by comparing the durations of these videos (16–20 min) to the time spans of V4V videos (up to 2 min), we did not exclude any video from the Operators dataset; instead, we neglected these useless windows since they do not have a huge impact on the predictions, especially since all the values underwent an averaging process to represent the value of blood pressure at every minute.

## 4. Results

While training the models there were many options to try in the feature extraction part. Based on what we obtained previously in Section 3.2.2, our work was focused on finding the best combination of different versions of EffecientNet and Resnet50V2 to include in the architecture of our models. In Table 3, we listed six (three SBP models and three DBP models) of our best models based on the MAE and mean accuracy. The mean accuracy was calculated by subtracting the predicted value from the actual value and taking the absolute value of the output and dividing it over the actual value to obtain the percentage of error. By applying this equation over all the test samples and taking the average and subtracting the output from 1, we obtain the mean accuracy. This metric is used to give a general idea of the accuracy of our predictions and decide whether the subject’s health condition is stable or may be at risk.

**Figure 6:**
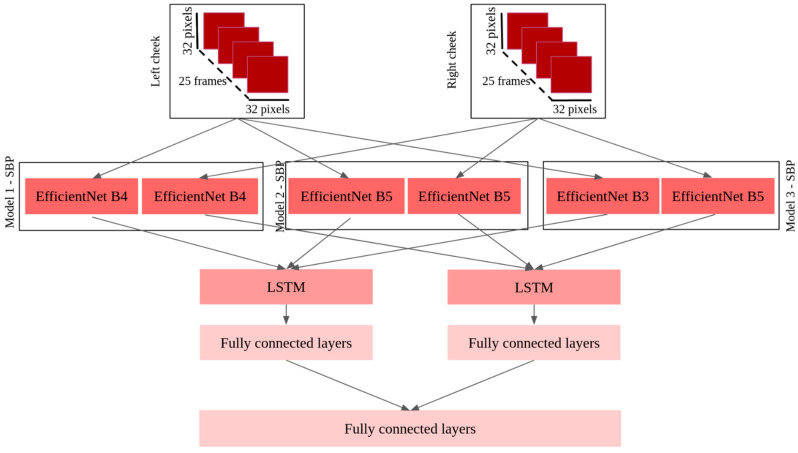
Architecture of the three best SBP models.

**Figure 7:**
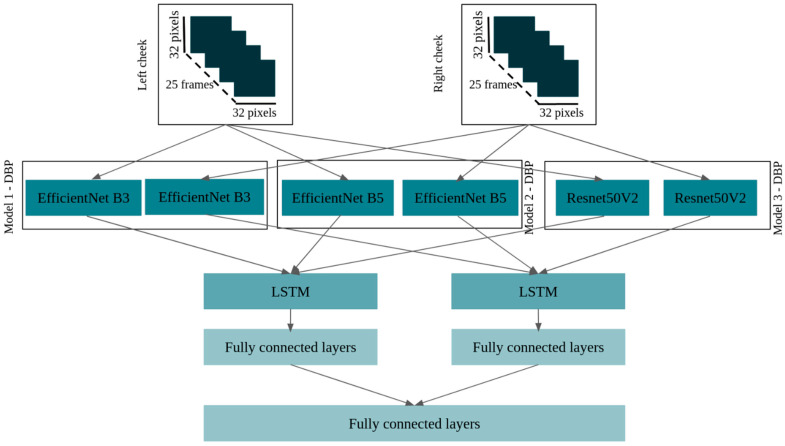
Architecture of the three best DBP models.

The architectures of these models are described in Figure 6 and Figure 7 for SBP models and DBP models, respectively.

Since we had a decent number of fine estimators and we were inspired by the ensemble learning concept [34], we decided to create some combinations of our models in order to have a better “learner” that can estimate blood pressure (SBP or DBP) with a lower MAE. By looking at Table 3, it is clear that the performances of the models are so close to each other based on the standards we went with; hence, our combinations were based on averaging the estimations of two models from the same type to satisfy the simplicity and, due to the ambiguity of determining the best model among the others, to have higher weight. Nevertheless, we list the best four combinations according to the lowest MAE and highest mean accuracy in Table 4.

To compare with other models, “V4V test set 1” has been changed due to the terms of compatibility with other models. Therefore, we gathered an objective set “V4V test set 2” that includes common and unusual values of SBP and DBP and satisfies the SNR condition for the models in [15]. The result came as it is shown in Table 5. The results indicate that three of our models performed better than all others regarding DBP, whereas one SBP model had a lower MAE and higher mean accuracy than the other models.

Regarding the correlation with respiratory rate, we tested our 10 models on the 60 videos of the Operator dataset (5 SBP models and 5 DBP models) and the other 4 models provided from [15] (these models predict SBP and DBP together). Therefore, as a result, we have 9 models that predict SBP and 9 models that predict DBP and, by averaging the output Pearson’s correlation coefficients over the 60 videos, we obtained the results shown in Table 6.

All five out of five SBP models have shown higher correlation than the other models, and four out of five DBP models had stronger correlation than all other models. Table 6 also shows that one SBP model and two DBP models had a high correlation with respiratory rate [32] (coefficient > 0.5 and *p*-value less than 0.05), which is strong evidence against the null hypothesis, as there is less than a 5% probability the null is correct. Therefore, we reject the null hypothesis and accept the alternative hypothesis that there is a relationship between the respiratory rate and the predicted blood pressure values obtained by our models, indicating a good response provided by our models, which were able to confirm the connection between blood pressure and respiratory rate more than the models we compared them with based on Pearson’s correlation coefficient.

By considering the results listed in the tables and the fact that we used “V4V test set 2” which contains new subjects from the V4V dataset, we would say that SBP Model_3 and DBP Model_1 are the best models for the following reasons: First, they almost performed the same on “V4V test set 1” and “V4V test set 2” with stable performance. Second, they both achieved Pearson’s correlation coefficients that indicated moderate to high correlation and were higher than the correlation coefficients that were obtained by the models we compared our performance with, and, finally, they are lighter to use if we decide to modify them to work through an application or website than using combinations that contain more than one trained model. There was a tradeoff decision to choose SBP Model_3 to be the best because we considered the stability in performance and gave it higher priority over the correlation, but on the other hand, the correlation coefficient of SBP Model_3 indicates a moderate correlation, which is not bad compared to the other models (e.g., SBP Model_2).

## 5. Discussion

This paper proposes a novel approach to estimate the blood pressure of subjects without the need for any equipment but the camera of any smartphone. Our methodology includes the extraction of ROIs from each frame in each video and passing these sequential images into a convolution neural network to obtain the spatial features. The outputs are then passed into LSTM to extract the temporal features within the image sequence. Our results indicate that changes in blood pressure can be detected from the right cheek and left cheek, which is reasonable due to the fact that these regions contain the main and largest blood vessels in the face [35]. By using these regions as inputs of our models, we were able to predict SBP and DBP with a decent MAE and acceptable mean accuracy considering the fact that no signal is extracted and no equipment but the camera is needed. Our models achieved this by determining the best pretrained models to use and the most useful regions of interest to extract, which we did before the training process began, including the procedure of upsampling the data, which let our models learn to estimate unusual values of SBP and DBP. We take into account that the SBP value is not related to the DBP value; hence, we trained our models separately, which means that we used the true SBP to train a part of the models, whereas the other part of the models was trained using the true DBP; therefore, there are no mutual weights or layers between the models that may affect the results and each model generates one value, which is either SBP or DBP based on the model.

The main challenge was the dataset; there is not enough diversity in the subjects’ skin color, since most of the subjects have light to medium color of skin, so our models may not be able to predict the blood pressure of subjects who have dark skin tones accurately. Another limitation was that the dataset is unbalanced. In other words, there is a lack of unusual values of SBP and DBP. We overcame this issue partially by upsampling the dataset, but even with this technique, our models have been trained on a limited range of features that indicate uncommon SBP or DBP, which may affect their estimations if they are being tested on subjects with unusual values of SBP or DBP where the filming or lighting conditions are different from the V4V dataset.

We also had a problem comparing our results with other models. This came from three factors: The first one is that no previous work has handled raw images as input like we did; instead, the main focus has been on extracting signals out of a cascade of frames, which made the extraction of the signal out of the videos a mandatory stage to perform the comparison. The second factor was that the datasets used in the papers were different with dissimilar ranges of the ground truth, since some papers created their models based on very limited ranges of SBP and DBP, while others had wider ranges based on which their models were built up. The final factor was the availability of the other models; most of the algorithms or models mentioned in the related work section are not available or published; hence, this was one major obstacle to achieving an objective comparison. Nevertheless, we were able to compare our results with models that have been trained over similar ranges of SBP and DBP to the ground truth of the V4V dataset. In general, some of our models had better performance than these models in estimating blood pressure, and their predictions achieved higher correlation with respiratory rate. This may be related to the fact that the CNN obtained the spatial features from all three channels, which have more useful information to estimate blood pressure than the signal extracted using the POS algorithm due to spatial averaging and other mathematical operations that may lead to loss of information, and in addition, the chosen threshold of SNR may have been not the best option for our dataset and it led to including many noisy signals from all our datasets. Regarding the inner comparison among our models, the MAE of the SBP models and the DBP models are very close, but we may attribute the small difference to the distributions of the training samples; as it is shown in Figure 2, the distribution of samples used to train the models to estimate DBP has a normal distribution even with upsampling the uncommon values; hence, we can say that SPB models are trained on a more generalized dataset than that on which DBP models were trained.

Concerning the idea of using Pearson’s correlation coefficient, we would like to mention that the metric is not new itself but using it to compare blood pressure values predicted by an algorithm or a model with another vital sign (respiratory rate in our case) has not executed before to the best to our knowledge.

Finally, we proposed in this paper a method that would give the subject an approximate value of the subject’s blood pressure with no need to attach any devices to his/her skin. This is not a method for medical use for diagnosis (e.g., hypertension or not). The main idea is to alarm the subject or responsible person (e.g., car park dispatcher) about fluctuations in his/her blood pressure that might indicate fatigue, stress, etc. However, our approach can be easily modified into an application, which will be extremely useful for blood pressure monitoring at home, school, office, or anywhere.

## 6. Conclusions

In this paper, we presented an innovative, inexpensive, and time-efficient method to estimate blood pressure using only a smartphone camera. Hybrid deep learning models including a convolutional neural network and LSTM were trained using cropped images of the right cheek and left cheek of the subjects of the V4V dataset. The results showed that our models are able to estimate blood pressure with a lower MAE and better performance than other published models. We also proposed a new criterion to evaluate our results by engaging the relation between blood pressure and respiratory rate in our testing process using our own dataset that provides the respiratory rate at every minute. We tested the correlation between the estimations of our models and the published ones with the respiratory rate. As a result, our models achieved a higher and stronger correlation than the models we compared our work with; hence, the task of estimating blood pressure has been completed according to the basic standards (i.e., MAE and mean accuracy) and our modern metric (i.e., Pearson correlation coefficient). In the future, we will try to extend the training set with subjects who are older or have darker skin tones with unfamiliar SBP and DBP values due to some medical conditions that the V4V dataset did not cover. In addition, our approach may be extended to estimate other vital signs such as heart rate, oxygen saturation, body temperature, etc.

## Figures and Tables

**Table 1:** Result of training different pretrained models.

Pretrained Model	RMSE	MAE
Xception	24.325	21.679
VGG16	21.142	17.178
Resnet50V2	17.427	14.883
InceptionV3	22.648	18.953
DenseNet121	22.358	18.267
EfficientNetB0	16.652	14.237
EfficientNetB1	16.333	14.006
EfficientNetB2	15.958	13.386

**Table 2:** Result of training EfficientNetB3 with 1 ROI as an input.

ROI	RMSE	MAE
Forehead	24.252	21.218
Left cheek	17.884	15.063
Right cheek	16.332	14.773

**Table 3:** Best models based on the lowest MAE and highest mean accuracy using “V4V test set 1”.

Model Type	Number of the Model	MAE	Mean Accuracy
SBP	Model_1	12.408	89.1%
	Model_2	14.491	87.3%
	Model_3	14.174	87.8%
DBP	Model_1	12.652	83.4%
	Model_2	11.371	85.2%
	Model_3	16.071	79.5%

**Table 4:** Best combinations based on the lowest MAE and highest mean accuracy.

Combination Type	Included Models	MAE	Mean Accuracy (%)
SBP	Model_1, Model_2	11.867	89.5%
	Model_1, Model_3	11.976	89.6%
DBP	Model_1, Model 2	10.706	86.2%
	Model_1, Model_3	12.499	84.1%

**Table 5:** Comparison between our models and available pretrained models using “V4V test set 2”.

Model Type	Number of the Model	MAE	Mean Accuracy (%)
SBP	AlexNet [16]	18.582	81.8%
	ResNet [17]	15.456	85.4%
	LSTM	14.579	86.7%
	Slapnicar et al. [18]	31.359	64.3%
	Combination (Model_1, Model 2)	17.477	84.7%
	Combination (Model_1, Model 3)	15.121	86.7%
	Model_1	18.084	83.7%
	Model_2	16.424	84.4%
	Model_3	13.749	88.2%
DBP	AlexNet [16]	13.460	83.1%
	ResNet [17]	12.528	83.9%
	LSTM	13.119	83.5%
	Slapnicar et al. [18]	20.621	62.1%
	Combination (Model_1, Model 2)	12.012	84.04%
	Combination (Model_1, Model 3)	11.169	84.9%
	Model_1	12.391	83.8%
	Model_2	12.697	83.02%
	Model_3	14.833	79.2%

**Table 6:** Comparison between our models and available pretrained models using Operators dataset.

Model Type	Number of the Model	Average Pearson’s Correlation Coefficient over 60 Videos	Average *p*-Value over 60 Videos
SBP	AlexNet [16]	0.4256	0.124
	ResNet [17]	0.4226	0.129
	LSTM	0.4189	0.133
	Slapnicar et al. [18]	-	-
	Combination (Model_1, Model 2)	0.4606	0.107
	Combination (Model_1, Model 3)	0.4863	0.092
	Model_1	0.4670	0.103
	Model_2	0.5028	0.048
	Model_3	0.4304	0.122
DBP	AlexNet [16]	0.4491	0.118
	ResNet [17]	0.4559	0.112
	LSTM	0.3920	0.154
	Slapnicar et al. [18]	-	-
	Combination (Model_1, Model 2)	0.5236	0.045
	Combination (Model_1, Model 3)	0.4623	0.106
	Model_1	0.5302	0.037
	Model_2	0.4743	0.097
	Model_3	0.4429	0.120

## Data Availability

Not applicable.
